# SLight-Net: a lightweight spectral-layer aware network for retinal disease detection based on optical coherence tomography

**DOI:** 10.3389/fcell.2026.1908247

**Published:** 2026-08-05

**Authors:** Yixiang Yao, Jin Hong, Rongli Zhang, Junhua Hu, Libin Lu, Peixin Tan, Zhongbiao Xu, Shuhua Luo

**Affiliations:** 1 School of Information Engineering, Nanchang University, Nanchang, China; 2 School of Artificial Intelligence, Nanchang University, Nanchang, China; 3 Department of Diagnostic Radiology, Li Ka Shing Faculty of Medicine, The University of Hong Kong, Pok Fu Lam, Hong Kong, China; 4 State Key Laboratory of Water Cycle and Water Security, China Institute of Water Resources and Hydropower Research, Beijing, China; 5 School of Information Engineering, Wuhan College, Wuhan, China; 6 Department of Radiotherapy, Guangdong Provincial People’s Hospital, Guangdong Academy of Medical Sciences, Southern Medical University, Guangzhou, China; 7 Ganzhou Key Laboratory of Medical Imaging and Artificial Intelligence, Medical Imaging Center, Ganzhou Institute of Medical Imaging, Ganzhou People’s Hospital, The Affiliated Ganzhou Hospital of Nanchang University, Ganzhou Hospital-Nanfang Hospital, Southern Medical University, Ganzhou, China

**Keywords:** anatomically informed priors, lightweight neural network, optical coherence tomography, retinal OCT classification, spectral-spatial attention

## Abstract

**Background:**

Retinal diseases are a major cause of preventable visual impairment, and optical coherence tomography (OCT) provides high-resolution cross-sectional imaging for retinal assessment. However, reliable interpretation of OCT B-scans remains challenging because of speckle noise, subtle layer-wise changes, and visually similar pathological patterns.

**Methods:**

We propose SLight-Net, a lightweight spectral-layer aware network for retinal OCT classification. The model is built on a compact three-stage convolutional backbone and incorporates two OCT-specific modules. The Frequency-Aware Spectral-Spatial Encoder (FASE) integrates local convolution, dilated contextual modeling, and learnable spectral modulation to capture multi-scale structural and frequency-aware retinal features. The Retinal Layer Depth Attention (RLDA) module further introduces a depth-direction anatomical prior to recalibrate feature responses along retinal layers. Deep supervision and exponential moving-average weight updating are used to improve optimization stability.

**Results:**

On the OCT-C8 benchmark, SLight-Net achieves 98.21% classification accuracy with only 1.204M parameters. Additional evaluation on OCT2017 shows 99.30% accuracy, suggesting that the model maintains stable performance under a different class setting while remaining compact.

**Conclusion:**

These findings indicate that OCT-specific spectral and layer-aware priors can support efficient retinal disease classification without relying on large generic backbones, providing a practical basis for lightweight computer-aided OCT analysis.

## 1 Introduction

**FIGURE 1:**
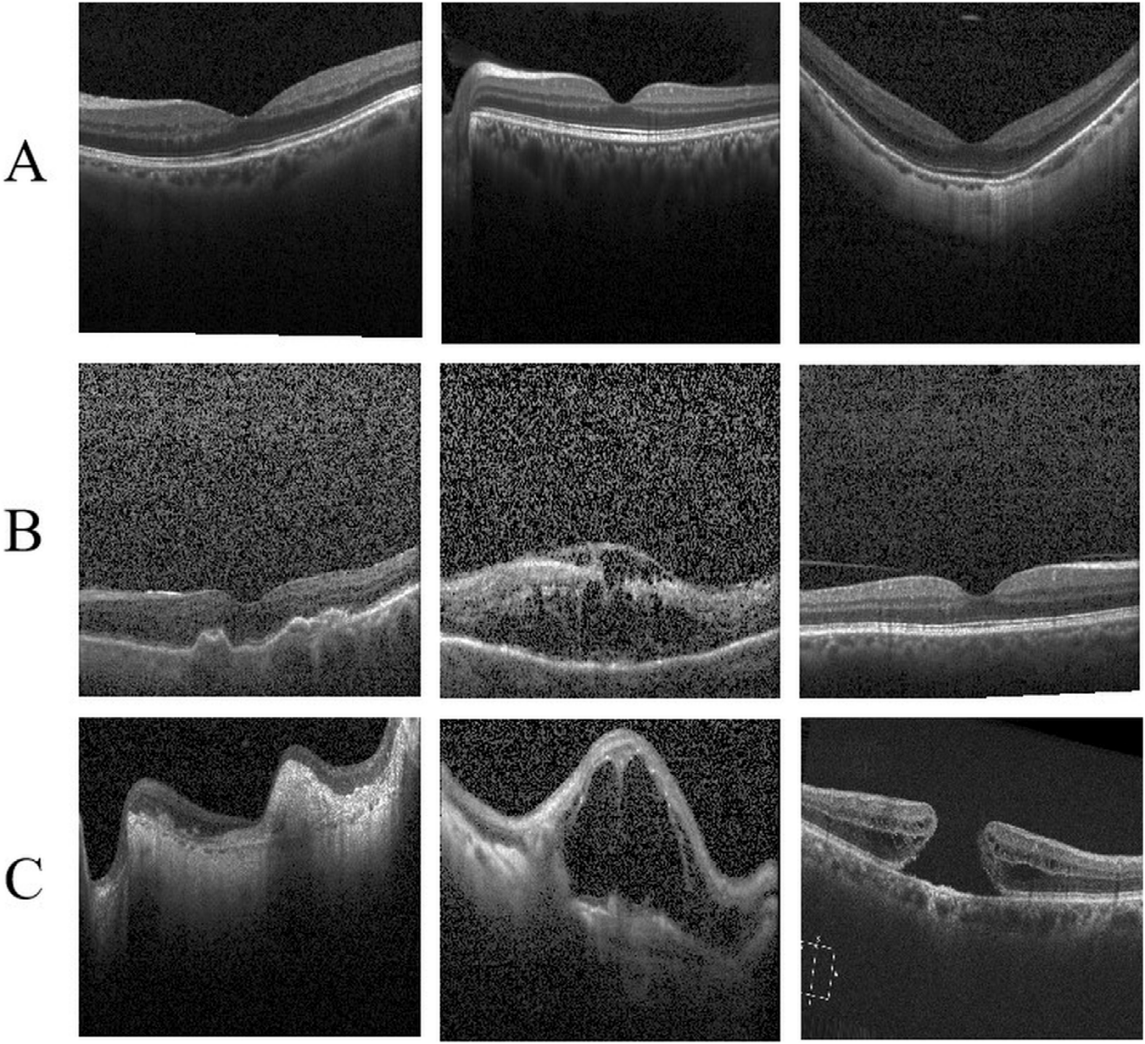
Representative OCT images illustrating major challenges in retinal disease classification. **(A)** Normal retinal samples with regular layered structures. **(B)** Low-quality OCT images affected by speckle noise and blurred retinal boundaries. **(C)** Diseased retinal samples showing diverse lesion morphology and structural deformation.

Retinal diseases remain among the major causes of irreversible visual impairment worldwide, although many of them are preventable or treatable when detected at an early stage. Common posterior-segment disorders, such as macular hole, central serous chorioretinopathy, diabetic retinopathy, diabetic macular edema, drusen-related age-related maculopathy, neovascular age-related maculopathy, and secondary choroidal neovascularization, may progress gradually before obvious symptoms occur. Once central visual function is affected, the resulting damage is often difficult to reverse. Optical coherence tomography (OCT) has become one of the most important non-invasive imaging modalities for posterior-segment examination, as it provides cross-sectional visualization of retinal structures at micrometer-level resolution (Bourne et al., 2021; Huang et al., 1991; Keane et al., 2012). However, the clinical benefit of OCT also brings a considerable reading burden. A routine examination can contain hundreds of B-scans, and accurate discrimination among visually similar retinal abnormalities often depends on the experience of trained ophthalmologists or retinal specialists. As illustrated in Figure 1, retinal OCT images may exhibit visually subtle normal-layer structures, quality degradation caused by speckle noise, and large morphological variations across diseased cases, all of which increase the difficulty of reliable image interpretation. This creates a practical need for reliable and efficient computer-aided OCT analysis.

To address this need, deep learning-based OCT image classification has been widely investigated in recent years. Early studies based on convolutional neural networks, including ResNet (He et al., 2016), DenseNet (Huang et al., 2017), and EfficientNet (Tan and Le, 2019), established strong baselines by learning discriminative retinal representations directly from OCT images. Subsequent studies further introduced hierarchical feature pyramids and multi-scale feature aggregation strategies (Sotoudeh-Paima et al., 2022; Peng et al., 2024) to improve the recognition of lesions with different sizes and morphologies. More recently, Vision Transformer-based models (Dosovitskiy et al., 2020)and hybrid CNN-Transformer architectures, such as HCTNet (Ma et al., 2022), Swin-Poly Transformer (He et al., 2023), and HTC-Retina (Laouarem et al., 2024), have been explored to enhance long-range dependency modeling while preserving local structural information. In parallel, frequency-aware retinal analysis has also attracted attention, as exemplified by WaveNet-SF (Cheng et al., 2026), which combines wavelet decomposition with spatial attention. These methods have substantially improved OCT classification performance, with several recent studies reporting accuracies approaching 99% on commonly used benchmark datasets under controlled experimental settings.

Despite these advances, several limitations remain. First, many high-performing approaches rely on relatively large generic backbones, often containing tens of millions of parameters. This limits their suitability for resource-constrained environments, such as primary-care ophthalmology clinics or edge-assisted screening systems. Second, although spectral or frequency-aware cues are informative for OCT images, where speckle noise, fine retinal boundaries, and subtle pathological textures coexist, existing methods often treat such information as a preprocessing operation or an auxiliary branch rather than integrating it into the main feature-extraction process. Third, OCT B-scans contain a strong anatomical prior: the retinal tissue is arranged in a layered structure along the depth direction. However, most current architectures process horizontal and vertical spatial dimensions in a largely isotropic manner and do not explicitly exploit this layer-wise regularity. These observations suggest that a compact model specifically designed around the spectral characteristics and laminar anatomy of retinal OCT may achieve competitive performance with a much smaller parameter budget than general-purpose backbones.

Motivated by this, we propose SLight-Net, a lightweight convolutional network for retinal OCT classification. The name SLight-Net indicates two key characteristics of the proposed model: it is lightweight, containing only 1.2M parameters, and it incorporates two OCT-specific design components represented by ‘S’ and ‘L’, namely, a spectral-aware component and a layer-aware component. Specifically, we design a Frequency-Aware Spectral-Spatial Encoder (FASE), which combines local convolution, dilated contextual modeling, and learnable spectral modulation through a soft selective fusion mechanism. This enables the network to adaptively balance local structural details and broader spectral-spatial representations within a unified feature block. In addition, we propose a Retinal Layer Depth Attention (RLDA) module, which introduces a learnable depth-direction prior and recalibrates intermediate feature maps according to layer-wise contextual dependencies. By embedding this anatomical regularity into the network, RLDA helps the model focus on retinal layer-related variations that are important for disease discrimination. The proposed modules are integrated into a compact three-stage CNN backbone with deep supervision and exponential moving-average weight averaging.

We evaluate SLight-Net primarily on the eight-class OCT-C8 dataset, which provides a fine-grained benchmark for retinal disease classification, and further conduct a supporting evaluation on the four-class OCT2017 dataset. The proposed model achieves 98.21% accuracy on OCT-C8 and 99.30% accuracy on OCT2017 while maintaining a substantially smaller parameter size than many recent high-performing models. Progressive ablation results on OCT-C8 verify the complementary contributions of FASE and RLDA, demonstrating that task-specific spectral and anatomical priors can provide an efficient alternative to simply scaling up generic network backbones.

The main contributions of this work are summarized as follows.

- We propose SLight-Net, a compact end-to-end retinal OCT classification framework with only 1.2M parameters, achieving competitive performance on two public OCT benchmarks while greatly reducing model complexity.
- We design the Frequency-Aware Spectral-Spatial Encoder (FASE), a tri-branch feature extraction block that integrates local convolution, dilated contextual modeling, and learnable spectral modulation through a soft selective fusion strategy.
- We introduce the Retinal Layer Depth Attention (RLDA) module, which explicitly incorporates a depth-direction anatomical prior and recalibrates retinal features according to layer-wise contextual information.
- We conduct comprehensive experiments, including comparisons with representative state-of-the-art methods and progressive ablation studies, to validate the effectiveness and parameter efficiency of the proposed framework.

## 2 Related work

### 2.1 Deep learning for retinal OCT classification

Early efforts to automate retinal OCT interpretation mainly relied on image-classification backbones pre-trained on natural images. Hassan et al. (2021) adapted AlexNet, ResNet18, and GoogLeNet to detect central serous retinopathy from denoised OCT scans, while Li et al. (Li et al., 2019) and Kim and Tran (2020) showed that ensembles based on ResNet50 variants could achieve strong performance in four-class OCT classification. In addition to directly transferring generic backbones, several studies introduced anatomy-aware preprocessing. For example, the layer-guided CNN proposed by Huang et al. (Huang et al., 2019) first segmented the inner limiting membrane and Bruch’s membrane using a pre-trained ReLayNet, and then fed cropped layer maps into parallel subnetworks for classification.

The development of Vision Transformers (Dosovitskiy et al., 2020) further promoted hybrid designs for OCT image analysis. HCTNet (Ma et al., 2022) combined a convolutional branch with a Transformer branch to fuse local and global representations. Based on the Swin Transformer backbone (Liu et al., 2021), Swin-Poly Transformer (He et al., 2023) introduced a polynomial cross-entropy objective and reported 97.11% accuracy on the eight-class OCT-C8 dataset. HTC-Retina (Laouarem et al., 2024) further integrated Transformer-based self-attention with hierarchical CNN features and achieved comparable performance on the same benchmark. These methods demonstrate the effectiveness of combining local convolutional features with long-range dependency modeling for OCT classification.

Multi-scale feature aggregation has also been widely explored. Iterative Fusion CNNs (Fang et al., 2019a) and multi-scale convolutional Networks (Sotoudeh-Paima et al., 2022) aggregate features across different resolutions, and later studies further refined such designs with denoising residual blocks (Peng et al., 2024). Other methods attempted to enhance lesion-relevant evidence more directly. The Edgen block proposed by (Karthik and Mahadevappa, 2023) replaced the conventional residual operator with a derivative-amplifying activation to emphasize retinal boundaries, while the lesion-aware network of (Fang et al., 2019b) used soft attention maps to highlight pathological regions. More recently, WaveNet-SF (Cheng et al., 2026) introduced wavelet-domain spatial attention and high-frequency feature compensation, reporting strong performance on both OCT-C8 and OCT 2017.

Overall, existing OCT classification methods have mainly improved performance through three directions: stronger generic backbones, multi-scale feature aggregation, and attention- or frequency-enhanced feature representation. However, many recent high-performing methods still rely on relatively large backbones, often containing tens of millions of parameters, which may reduce deployment efficiency in resource-constrained screening scenarios. Moreover, the layered anatomy of the retina, one of the most distinctive structural properties of OCT B-scans, has usually been incorporated only indirectly, either through explicit pre-segmentation or generic spatial attention. These observations motivate the design of a compact OCT-specific network that integrates both spectral-spatial representation and retinal layer priors.

### 2.2 Frequency-aware and spectral-spatial learning

Frequency-aware representations have long been used to describe textures, noise patterns, and structural variations in visual signals. Their integration into deep neural networks has recently attracted increasing attention. GFNet (Rao et al., 2021) replaced self-attention with a learnable global filter in the Fourier domain and demonstrated competitive ImageNet performance with reduced computational cost. FcaNet (Qin et al., 2021) extended squeeze-and-excitation channel attention by replacing global average pooling with discrete cosine transform components, showing that frequency-domain summaries can provide more informative channel descriptors than purely spatial statistics. The Frequency Selection Network (FSNet) (Cui et al., 2024) adaptively decomposed intermediate features into frequency subbands and reweighted them for image restoration tasks. In low-level vision tasks, FFTformer (Kong et al., 2023) reformulated spatial attention as element-wise multiplication in the Fourier domain. For retinal OCT analysis, WaveNet-SF (Cheng et al., 2026) decomposed B-scans into low- and high-frequency subbands and processed them with dedicated branches. Beyond retinal OCT classification, frequency-spatial synergy has also been explored in other medical imaging tasks. FSS-Net (Liu J. et al., 2026) introduced a Frequency-Spatial Synergy Network with wavelet attention for carotid artery ultrasound segmentation, showing that frequency-domain cues and spatial-domain representations can complement each other in noise-sensitive medical images. This observation is consistent with the motivation of frequency-aware OCT representation learning, since OCT B-scans also contain speckle noise, subtle boundary variations, and fine pathological textures.

These studies suggest that frequency-aware information can provide useful complementary cues for medical image analysis, especially for OCT images where speckle noise, fine laminar boundaries, and subtle pathological textures coexist. However, existing OCT methods often introduce spectral or frequency-related information as a preprocessing operation or as an auxiliary pathway separated from the main feature-extraction backbone. In contrast, the proposed Frequency-Aware Spectral-Spatial Encoder (FASE) integrates learnable spectral modulation with local convolution and dilated contextual modeling within the core feature-extraction process. This design allows spatial and spectral-spatial cues to be adaptively fused rather than being treated as isolated sources of information.

### 2.3 Attention mechanisms and anatomical priors in OCT

Attention mechanisms have become an important component of modern recognition networks. Squeeze-and-Excitation (SE) (Hu et al., 2018) recalibrates channel responses through global pooling and a lightweight multilayer perceptron, while the Convolutional Block Attention Module (CBAM) (Woo et al., 2018) and the Bottleneck Attention Module (BAM) (Park et al., 2018) extend attention modeling to the spatial dimension using convolutional operations. More recent attention variants further explore non-local interactions, spatial recalibration, and efficient context modeling. These mechanisms have also been widely adopted in medical image analysis, where they are commonly used to emphasize informative regions and suppress irrelevant background responses.

However, attention modules are often used as generic plug-in components and may not fully exploit the anatomical structure of a specific imaging modality. Retinal OCT provides a particularly clear example. Each B-scan is acquired along a well-defined depth direction, and the retina exhibits a relatively stable laminar organization across subjects. This vertical layer-wise arrangement contains important diagnostic information, because many retinal diseases are associated with disruptions, thickening, fluid accumulation, or abnormal reflectivity within specific retinal layers. Nevertheless, most existing OCT models process the horizontal and vertical dimensions in a largely symmetric manner, leaving the model to infer layer-wise regularity implicitly from training data.

The proposed Retinal Layer Depth Attention (RLDA) module is designed to address this limitation by incorporating depth-aligned information directly into the feature representation. Instead of treating retinal layer organization as an implicit pattern, RLDA models contextual interactions along the retinal depth axis and recalibrates features according to layer-wise dependencies. In this way, the laminar structure of OCT is treated as an explicit architectural prior, complementing the spectral-spatial modeling introduced by FASE.

Recent medical imaging studies also suggest that model design can benefit from explicitly incorporating task-specific structural or clinical priors. DGSSA (Liu B. et al., 2026) improves retinal vessel segmentation domain generalization by combining structural and stylistic augmentation, highlighting the importance of anatomical structure variation in retinal image analysis. ClinReadNet (Xiao et al., 2026) further introduces a clinical reading-inspired network for low-dose abdominal CT image quality assessment, indicating that clinical reasoning patterns can also guide medical model design. Although these studies address different tasks, they share a similar principle: medical imaging models should not only rely on generic visual backbones, but should also exploit modality-specific structures and task-related prior knowledge.

## 3 Methods

### 3.1 Overall architecture of SLight-Net

**FIGURE 2:**
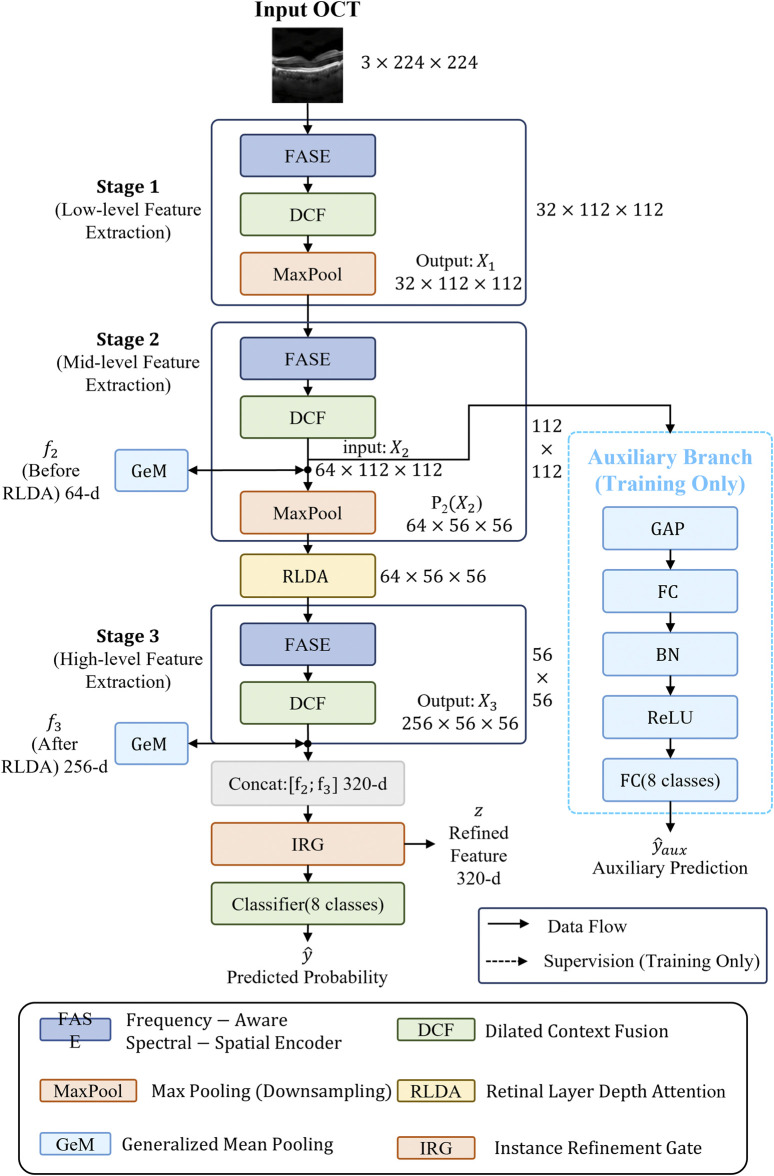
Overall framework of SLight-Net. The figure shows the three-stage CNN backbone, FASE and DCF units, the RLDA module after the second pooling operation, the Stage-2 and Stage-3 feature fusion path, IRG, the final classifier, and the auxiliary branch used during training.

The overall framework of SLight-Net is shown in Figure 2. The network is designed as a compact three-stage convolutional architecture for retinal OCT classification. For an input OCT B-scan resized to 224 × 224, the model first extracts low-level representations through the first FASE-based convolutional stage. The network then gradually builds mid-level layer-boundary features and high-level disease-discriminative representations through three successive stages. Unlike heavy generic backbones, SLight-Net uses only lightweight convolutional units and contains 1.204M parameters in the final configuration.

According to the actual implementation, Stage 1 maps the input image to 32 channels and performs the first downsampling by max pooling. Stage 2 further expands the feature dimension to 64 channels, and its output is used in two ways: one path is retained as an unmodified mid-level feature for later feature fusion, and the other path is downsampled and sent to the Retinal Layer Depth Attention (RLDA) module. For a 224 × 224 input, the feature map entering RLDA has 64 channels and a spatial size of 56 × 56. Stage 3 then processes the RLDA-enhanced feature and outputs a 256-channel high-level representation. This design allows the classifier to combine unmodified mid-level features before RLDA with depth-aware high-level features after RLDA.

The first stage of SLight-Net can be expressed by Equation 1. It contains a FASE block, a DCF unit, and a max-pooling operation for spatial downsampling.

$$X_{1}=P_{1}\left(D_{1}\left(E_{1}\left(X\right)\right)\right)$$ (1)

where X denotes the input OCT image, E_1_ (·) denotes the first FASE block, D_1_ (·) denotes the first DCF unit, P_1_ (·) denotes max pooling, and X_1_ denotes the output feature of Stage 1. This equation describes the initial feature extraction and the first spatial downsampling process.

The second stage extracts a 64-channel mid-level representation. This feature contains rich retinal texture and layer-boundary information and is therefore kept for later multi-level fusion.

The second-stage feature extraction is expressed by Equation 2.

$$X_{2}=D_{2}\left(E_{2}\left(X_{1}\right)\right)$$ (2)

where E_2_ (·) and D_2_ (·) denote the FASE and DCF units in Stage 2, respectively, and X_2_ represents the Stage-2 feature before RLDA. In the final model, X_2_ has a spatial size of 112 × 112 before the second pooling operation.

After the Stage-2 feature is obtained, it is downsampled and then processed by RLDA. This step explicitly introduces retinal depth-axis modeling while still preserving sufficient spatial resolution.

The RLDA-enhanced feature is calculated using Equation 3.

$$\tilde{X_{2}}=R\left(P_{2}\left(X_{2}\right)\right)$$ (3)

where P_2_ (·) denotes the second max-pooling operation, R (·) denotes the RLDA module, and $\tilde{X_{2}}$ is the RLDA-enhanced feature. For a 224 × 224 input, P_2_ (X_2_) has 64 channels and a spatial size of 56 × 56.

The RLDA-enhanced feature is then fed into Stage 3 to extract high-level disease-related semantic features.

The calculation of the third stage is formulated as Equation 4.

$$X_{3}=D_{3}\left(E_{3}\left(\tilde{X_{2}}\right)\right)$$ (4)

where E_3_ (·) and D_3_ (·) denote the FASE and DCF units in Stage 3, respectively, and X_3_ denotes the final convolutional feature map before global pooling.

SLight-Net uses both Stage-2 and Stage-3 features for classification. The Stage-2 feature is pooled before RLDA, whereas the Stage-3 feature is taken from the RLDA-enhanced path. This asymmetry is intentional: the former preserves unmodified mid-level retinal textures, while the latter provides depth-aware high-level representation.

The pooled Stage-2 and Stage-3 features are calculated using Equation 5.

$$f_{2}=G\left(X_{2}\right),f_{3}=G\left(X_{3}\right)$$ (5)

where G (·) denotes GeM pooling, f_2_ is the pooled Stage-2 feature extracted before RLDA, and f_3_ is the pooled Stage-3 feature extracted after RLDA enhancement. The two features are complementary in that f_2_ emphasizes original layer-boundary textures, while f_3_ contains depth-aware semantic information.

Finally, the two feature vectors are concatenated and refined by the Instance Refinement Gate (IRG) before being sent to the classifier.

$$z=I\left(\left[f_{2};f_{3}\right]\right)$$ (6)

$$\hat{y}=\text{Softmax}\left(W_{c}z+b_{c}\right)$$ (7)

where [·; ·] denotes feature concatenation, I (·) denotes IRG, z is the refined feature vector, W_c_ and b_c_ are the weight matrix and bias of the final classifier, and ŷ denotes the predicted class probability distribution. Equations 6, 7 describe the final feature fusion and classification process.

During training, an auxiliary classifier is attached to X_2_ to provide intermediate supervision. This branch is used only during training and is removed during inference. In addition, an exponential moving average (EMA) copy of the model weights is maintained for evaluation to improve prediction stability.

### 3.2 Frequency-Aware Spectral-Spatial Encoder

**FIGURE 3:**
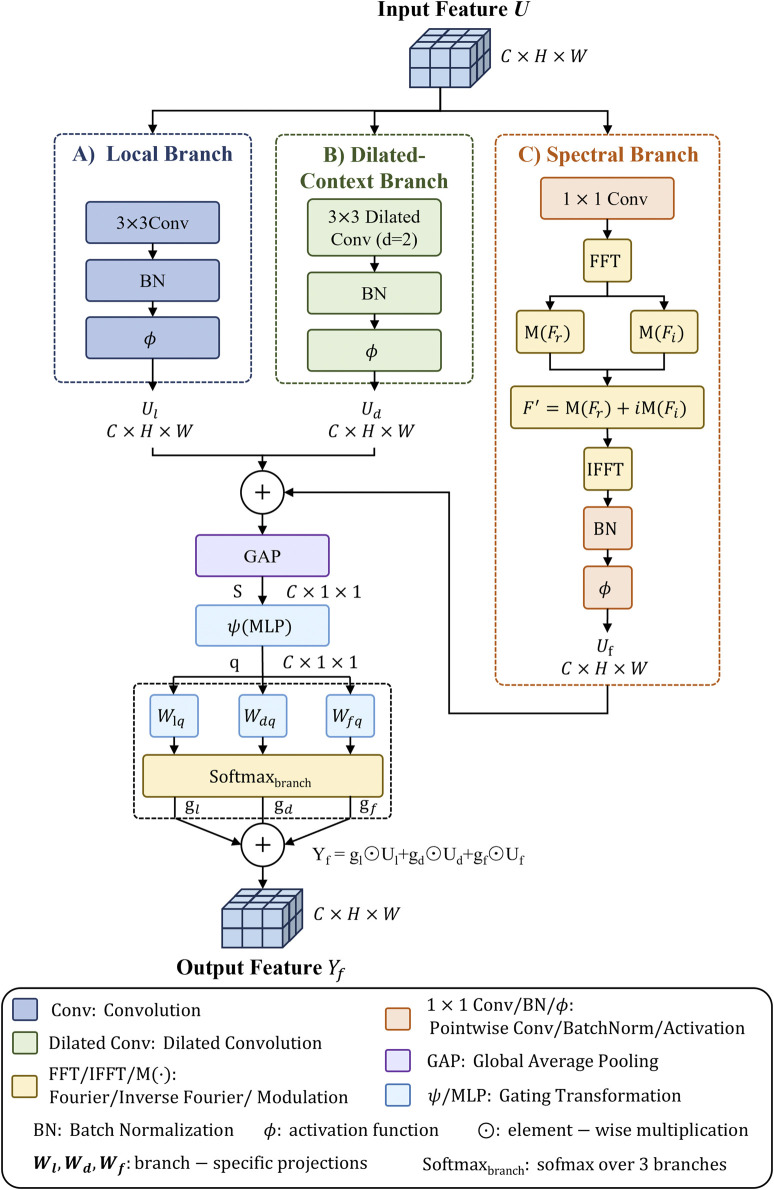
Structure of FASE. The figure shows the local (3 × 3) convolution branch, the dilated (3 × 3) convolution branch, the Fourier-domain spectral branch, and the selective fusion gate.

The detailed structure of the Frequency-Aware Spectral-Spatial Encoder is shown in Figure 3. FASE is the main feature extraction unit of SLight-Net and is used in all three convolutional stages. It is designed based on the observation that retinal OCT images contain useful information in multiple forms. Local retinal boundaries and lesion margins can be effectively captured by standard spatial convolution; broader anatomical context requires an enlarged receptive field; and subtle layer textures, speckle-related patterns, and fine structural variations can be further described through frequency-aware modulation. Therefore, FASE contains three parallel branches: a local branch, a dilated-context branch, and a spectral branch.

Given an input feature map (U), the local branch uses a standard (3 × 3) convolution followed by batch normalization and activation. This branch mainly focuses on local retinal edges, lesion boundaries, and fine texture details. The local feature extraction process is shown in Equation 8.

$$U_{l}=\phi \left(\text{BN}\left(C_{3\times 3}\left(U\right)\right)\right)$$ (8)

where U denotes the input feature map of FASE, U_l_ denotes the output of the local branch, C_3×3_ (·) denotes a standard 3 × 3 convolution, BN(·) denotes batch normalization, and ϕ (·) denotes the activation function. This branch preserves fine spatial details through local convolution.

The dilated-context branch uses a (3 × 3) convolution with dilation rate 2. Compared with the local branch, it enlarges the effective receptive field without introducing additional downsampling, allowing the network to capture broader structural patterns in OCT images. This process is formulated as Equation 9.

$$U_{d}=\phi \left(\text{BN}\left(C_{3\times 3}^{d=2}\left(U\right)\right)\right)$$ (9)

where ( $U_{d}$ ) denotes the output of the dilated-context branch, and ( $C_{3\times 3}^{d=2}\left(\cdot \right)$ ) denotes a (3 × 3) convolution with dilation rate 2. This branch complements the local branch by aggregating a wider spatial context while preserving the spatial resolution of the feature map.

The spectral branch first maps the input feature to the target channel dimension using a (1 × 1) convolution. The projected feature is then transformed into the Fourier domain. In accordance with the final implementation, the real and imaginary components are separately processed by the same lightweight convolutional modulation function and then recombined before the inverse Fourier transform. The projection operation is given by Equation 10.

$$\tilde{U_{}}=C_{1\times 1}\left(U\right)$$ (10)

where ( $C_{1\times 1}\left(\cdot \right)$ ) denotes a (1 × 1) projection convolution, and ( $\tilde{U_{}}$ ) is the projected feature map used as the input to the spectral branch.

After projection, the feature map is transformed into the Fourier domain, as shown in Equation 11.

$$F=\text{FFT}\left(\tilde{U_{}}\right)=F_{r}+iF_{i}$$ (11)

where ( $\text{FFT}\left(\cdot \right)$ ) denotes the two-dimensional Fourier transform, ( F ) is the complex Fourier representation, and ( $F_{r}$ ) and ( $F_{i}$ ) denote its real and imaginary components, respectively.

The real and imaginary components are then modulated separately using the same lightweight convolutional modulation function. This operation is expressed by Equation 12.

$$F^{\prime }=M\left(F_{r}\right)+iM\left(F_{i}\right)$$ (12)

where ( $M\left(\cdot \right)$ ) denotes the shared lightweight convolutional modulation function applied separately to the real and imaginary components. This equation describes the learnable frequency-aware modulation in the spectral branch.

Finally, the modulated Fourier representation is transformed back into the spatial domain, followed by batch normalization and activation, as shown in Equation 13.

$$U_{f}=\phi \left(\text{BN}\left(\text{IFFT}\left(F^{\prime }\right)\right)\right)$$ (13)

where ( $\text{IFFT}\left(\cdot \right)$ ) denotes the inverse Fourier transform, and ( $U_{f}$ ) is the spatial-domain output of the spectral branch. In mixed-precision training, the Fourier branch is computed in single precision to improve numerical stability.

After the three branches are computed, FASE uses a selective fusion mechanism to adaptively assign different weights to the local, contextual, and spectral representations. First, the three branch outputs are summed and globally pooled to generate a compact descriptor, as shown in Equation 14.

$$s=\text{GAP}\left(U_{l}+U_{d}+U_{f}\right)$$ (14)

where ( $\text{GAP}\left(\cdot \right)$ ) denotes global average pooling, and (s) denotes the global descriptor used for branch selection. This descriptor summarizes the overall response of the three branches and provides global information for adaptive fusion.

The descriptor is then passed through a lightweight gating network to generate branch-wise weights. Specifically, the gating network first transforms (s) into a compact hidden representation, and then produces three groups of channel-wise weights corresponding to the local, dilated-context, and spectral branches. The weights are normalized by softmax across the branch dimension, as shown in Equation 15.

$$\left[g_{l},g_{d},g_{f}\right]=\text{Softmax}\left(\left[W_{l}q;W_{d}q;W_{f}q\right]\right),q=\psi \left(s\right)$$ (15)

where (q) denotes the hidden representation generated from the global descriptor, ( $\psi \left(\cdot \right)$ ) denotes the lightweight transformation in the gating network, ( $W_{l}$ ), ( $W_{d}$ ), and ( $W_{f}$ ) are learnable projections for the three branches, and ( $g_{l}$ ), ( $g_{d}$ ), and ( $g_{f}$ ) denote the weights of the local, dilated-context, and spectral branches, respectively. The softmax operation ensures that the three branch contributions are adaptively normalized.

Finally, the three branch outputs are combined by weighted summation to obtain the output of FASE, as shown in Equation 16.

$$Y_{f}=g_{l}⊙U_{l}+g_{d}⊙U_{d}+g_{f}⊙U_{f}$$ (16)

where ( $Y_{f}$ ) denotes the output feature of FASE, and ⊙ denotes channel-wise multiplication with broadcasting along the spatial dimensions. Equation 16 indicates that FASE does not simply concatenate all branch outputs. Instead, it selectively integrates local spatial details, broader contextual information, and spectral-spatial cues according to the learned branch importance.

Compared with traditional selective-kernel mechanisms that mainly select among spatial kernels, FASE performs adaptive selection among spatially local, spatially contextual, and frequency-aware representations. This selective fusion summarizes the three branch outputs to generate branch-wise weights, and the final feature is obtained by weighted summation rather than direct concatenation. This design keeps the module compact while allowing each OCT image to adaptively emphasize local retinal details, wider anatomical context, or frequency-aware texture cues.

### 3.3 Retinal Layer Depth Attention

**FIGURE 4:**
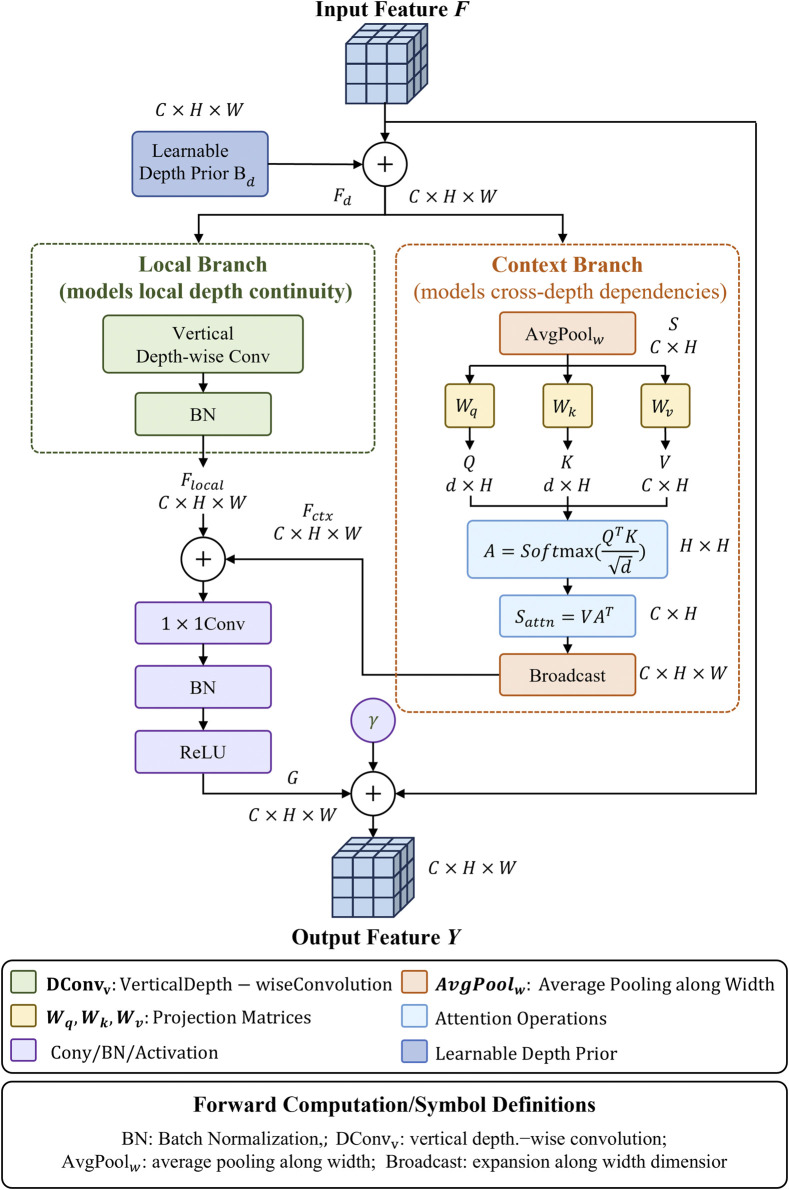
Structure of RLDA. The figure shows the learnable depth prior, the vertical depth-wise convolution branch, the cross-depth attention branch, the feature fusion operation, and the residual connection with a learnable scaling coefficient.

The detailed structure of the Retinal Layer Depth Attention (RLDA) module is shown in Figure 4. RLDA is the second core module of SLight-Net and is designed to exploit the anatomical structure of retinal OCT images. Unlike natural images, OCT B-scans have a meaningful vertical depth direction. Retinal tissues are arranged in layered anatomical structures, and many retinal diseases are associated with abnormalities in specific layers, such as local disruption, thickening, fluid accumulation, or abnormal reflectivity. A standard two-dimensional convolution treats the vertical and horizontal axes in the same way, which may not fully exploit this anatomical regularity. Therefore, RLDA introduces depth-aware modeling into the CNN feature representation.

In the final architecture, RLDA is inserted after the second-stage pooling operation. This placement is deliberate. If the module is inserted too early, the feature maps are dominated by low-level edges and local textures and may not contain sufficient disease-related semantic information. If the module is applied after global pooling or at the final classification stage, the explicit spatial arrangement along the retinal depth axis would already be lost. Therefore, RLDA is placed before Stage 3, allowing depth-aware recalibration to influence the subsequent high-level feature extraction process. The Stage-2 output (X2) has 64 channels and a spatial size of (112 × 112). After the second max-pooling operation, the feature map entering RLDA has 64 channels and a spatial size of (56 × 56) for a (224 × 224) input image.

Let ( $F\in R^{C\times H\times W}$ ) denote the input feature map to RLDA, where (C) (H), and (W) represent the channel number, height, and width, respectively. RLDA first introduces a learnable depth prior ( $B_{d}$ ). This prior is stored with a maximum height and is sliced or interpolated to match the current feature height. It is then broadcast along the width dimension and added to the input feature. This process can be expressed by Equation 17.

$$F_{d}=F+B_{d}$$ (17)

where ( $B_{d}\in R^{C\times H\times 1}$ ) denotes the learnable depth prior after height matching, and ( $F_{d}$ ) denotes the feature map enhanced by the depth prior. Equation 17 provides the model with a trainable position-dependent bias along the retinal depth axis. This operation does not force a hard segmentation of retinal layers; instead, it introduces a soft anatomical prior that allows different channels to respond differently at different depths.

After adding the depth prior, RLDA applies a vertical depth-wise convolution to model local dependencies along the retinal depth direction. The convolution is performed channel by channel with a vertically oriented kernel, so it captures local continuity among neighboring retinal depths while keeping the parameter cost low. The local depth-wise modeling process is shown in Equation 18.

$$F_{local}=\text{BN}\left(\text{DCon}v_{v}\left(F_{d}\right)\right)$$ (18)

where ( $\text{DCon}v_{v}\left(\cdot \right)$ ) denotes the vertical depth-wise convolution and ( $F_{local}$ ) denotes the local depth-aware feature. Since the convolution is depth-wise, it focuses on local depth continuity without mixing all channels at this step.

To further capture longer-range dependencies along the depth axis, RLDA constructs a one-dimensional depth sequence by averaging the feature map along the width dimension. This operation compresses lateral variations and summarizes each retinal depth position using the average response across the horizontal direction. The depth sequence is obtained by Equation 19.

$$S=\text{AvgPoo}l_{w}\left(F_{d}\right)$$ (19)

where ( $\text{AvgPoo}l_{w}\left(\cdot \right)$ ) denotes average pooling along the width dimension, and ( $S\in R^{C\times H}$ ) denotes the resulting depth sequence. Equation 19 converts the two-dimensional feature map into a compact depth-wise representation while preserving the ordering of retinal layers along the vertical axis.

Based on the depth sequence, RLDA generates query, key, and value representations using lightweight one-dimensional projections. This process is formulated as Equation 20.

$$Q=W_{q}S,K=W_{k}S,V=W_{v}S$$ (20)

where ( $W_{q}$ ), ( $W_{k}$ ), and ( $W_{v}$ ) denote the learnable projection functions for query, key, and value, respectively. (Q) and (K) are projected into a hidden dimension (d), while (V) retains the channel dimension used for reconstructing the depth-aware context. These projections prepare the depth sequence for cross-depth attention.

The cross-depth attention matrix is then computed along the height dimension. This attention operation models the relationship between different retinal depth positions, enabling the network to capture long-range dependencies among non-adjacent layers. The attention calculation is expressed by Equation 21.

$$A=\text{Softmax}\left(Q^{T}K/\sqrt{d}\right)$$ (21)

where ( $A\in R^{H\times H}$ ) denotes the cross-depth attention matrix, and (d) denotes the hidden dimension of the query and key projections. Equation 21 calculates the similarity between different depth positions and normalizes the result to obtain attention weights.

The depth-aware context is generated by applying the attention matrix to the value representation, as shown in Equation 22.

$$S_{attn}=VA^{T}$$ (22)

where ( $S_{\text{attn}}\in R^{C\times H}$ ) denotes the attention-enhanced depth sequence. This sequence contains long-range contextual information across the retinal depth axis.

The one-dimensional depth context is then broadcast back to the original two-dimensional spatial shape so that it can be fused with the local depth-wise feature. This process is shown in Equation 23.

$$F_{ctx}=\text{Broadcast}\left(S_{attn}\right)$$ (23)

where ( $\text{Broadcast}\left(\cdot \right)$ ) denotes expansion along the width dimension, and ( $F_{\text{ctx}}\in R^{C\times H\times W}$ ) denotes the restored depth-aware context feature. Equation 23 maps the cross-depth context back to the spatial feature representation.

After obtaining both the local depth-wise feature and the long-range depth-aware context, RLDA fuses them through a (1 × 1) projection, batch normalization, and activation. This operation is formulated as Equation 24.

$$G=\text{ReLU}\left(\text{BN}\left(C_{1\times 1}\left(F_{local}+F_{ctx}\right)\right)\right)$$ (24)

where ( $C_{1\times 1}\left(\cdot \right)$ ) denotes a (1 × 1) convolution, and (G) denotes the fused depth-aware feature. Equation 24 combines local depth continuity and long-range layer dependency into a unified feature representation.

Finally, RLDA uses a residual connection with a learnable scaling coefficient to produce the module output. The final output is given by Equation 25.

Y=F+γG (25)

where ( γ ) denotes a learnable scaling coefficient, and (Y) denotes the output feature of RLDA. This residual design allows RLDA to introduce depth-aware information while preserving the original feature representation. The scaling coefficient controls the strength of the depth-aware residual branch during training.

Because the attention operation is computed only over (H) depth positions after lateral aggregation, rather than over all (H × W) spatial positions, RLDA remains computationally lightweight. It does not replace the convolutional backbone; instead, it supplements the backbone with an OCT-specific depth-axis prior. Together with FASE, the two modules form a complementary pair: FASE captures local, contextual, and frequency-aware texture cues, while RLDA introduces retinal layer organization along the depth axis. As shown in the progressive ablation study in Section 4.5, adding FASE improves the accuracy from 97.96% to 98.11%, and further introducing RLDA increases the accuracy to 98.21%. These results indicate that FASE provides the main spectral-spatial representation enhancement, while RLDA contributes additional depth-aware modeling based on retinal laminar structure. In this way, SLight-Net achieves a compact OCT-specific representation without substantially increasing the model scale.

### 3.4 Auxiliary supervision, instance refinement, and training strategy

Although SLight-Net is mainly designed around FASE and RLDA, several lightweight supporting strategies are adopted in the final implementation to improve optimization stability and refine the final feature representation. These components are not treated as the main contributions of this work, but they are included in the method description because they are part of the final SLight-Net architecture.

First, an auxiliary classification head is attached to the second-stage feature map (X_2_) before the second max-pooling operation. This branch consists of adaptive average pooling, a linear layer, batch normalization, ReLU activation, and a final linear classifier. It provides direct supervision to the mid-level representation, encouraging Stage 2 to learn discriminative retinal texture and layer-boundary features rather than serving only as a transitional feature extractor. During inference, the auxiliary branch is removed and only the main classification path is used, so it does not increase the deployment cost.

The auxiliary prediction is generated by Equation 26.

$$\hat{y_{aux}}=H_{aux}\left(X_{2}\right)$$ (26)

where ( $H_{\text{aux}}\left(\cdot \right)$ ) denotes the auxiliary classification head, ( $X_{2}$ ) denotes the second-stage feature map before RLDA, and $\left(\hat{y_{\text{aux}}}\right)$ denotes the auxiliary prediction. This equation describes how intermediate supervision is derived from the Stage-2 representation.

The auxiliary loss is then calculated by Equation 27.

$$L_{aux}=\text{CE}\left(\hat{y_{aux}},y\right)$$ (27)

where (y) denotes the ground-truth label, and $\left(\text{CE}\left(\cdot \right)\right)$ denotes the cross-entropy loss. This loss provides an additional optimization signal for the intermediate feature map during training.

Second, SLight-Net uses an Instance Refinement Gate (IRG) after the multi-level feature concatenation step. After GeM pooling, the pre-RLDA Stage-2 feature ( $f_{2}$ ) and the post-RLDA Stage-3 feature ( $f_{3}$ ) are concatenated into a 320-dimensional vector ( $z_{0}$ ). IRG first generates an instance-specific channel attention vector, as shown in Equation 28.

$$a=\sigma \left(W_{2}\delta \left(W_{1}z_{0}\right)\right)$$ (28)

where ( $W_{1}$ ) and ( $W_{2}$ ) denote learnable linear transformations, $\left(\delta \left(\cdot \right)\right)$ denotes the ReLU activation function, $\left(\sigma \left(\cdot \right)\right)$ denotes the sigmoid function, and (a) denotes the instance-specific channel gate. This operation allows the model to estimate the relative importance of different feature dimensions for each input sample.

The gated feature is then processed by a lightweight fusion transformation and added back to the original feature vector, as formulated in Equation 29.

$$z=z_{0}+\Phi \left(z_{0}⊙a\right)$$ (29)

where ⊙ denotes element-wise multiplication, $\Phi \left(\cdot \right)$ denotes the fusion transformation implemented by a linear layer, batch normalization, and ReLU activation, and (z) denotes the refined feature vector. Equation 29 shows that the gated representation is further transformed and fused with the original feature through a residual connection.

The final classifier is implemented as a linear layer. Given the refined feature vector (z), the main prediction is obtained by Equation 30, which restates Equation 7 for completeness.

$$\hat{y}=\text{Softmax}\left(W_{c}z+b_{c}\right)$$ (30)

where ( $W_{c}$ ) and ( $b_{c}$ ) denote the weight matrix and bias of the final classifier, respectively, and $\left(\hat{y}\right)$ denotes the predicted class probability distribution.

The main classification loss is defined in Equation 31.

$$L_{main}=\text{CE}\left(\hat{y},y\right)$$ (31)

The final training objective combines the main classification loss and the auxiliary loss, as shown in Equation 32.

$$L=L_{main}+\alpha_{auxL_{aux}}$$ (32)

where $\left(\alpha_{\text{aux}}\right)$ is the weight of the auxiliary loss and is set to 0.4 in the final training configuration. No additional prototype-based or contrastive loss is introduced in the final SLight-Net model.

Training is conducted using a two-stage optimization strategy. In the first stage, the model is trained with the AdamW optimizer, cosine learning-rate scheduling, label-smoothed cross-entropy, horizontal flipping, and small-angle affine augmentation. An exponential moving average (EMA) copy of the model weights is maintained during training with a decay factor of 0.999 and is used for validation and final evaluation. In the second stage, the best model from the first stage is further fine-tuned with a lower learning rate of ( $1\times 10^{-5}$ ), while Batch Normalization statistics are frozen. This stage is used to stabilize the learned feature distribution and refine the final decision boundary without changing the network architecture. Additional implementation details, including data augmentation, optimizer settings, EMA, batch size, AMP training, and the single-precision FFT implementation, are provided in Section 4.3.

To avoid numerical instability in Fourier-domain operations, the spectral branch in FASE is forced to single precision during FFT and inverse FFT computation. The final reported SLight-Net model uses FASE and RLDA as its two OCT-specific core modules, with DCF, auxiliary supervision, IRG, EMA, and two-stage optimization serving as lightweight supporting components.

## 4 Experiments and results

### 4.1 Dataset

In this study, we evaluated SLight-Net on two public retinal OCT datasets: the eight-class RetinalOCT dataset, also known as OCT-C8 (Naren, 2021), and the four-class OCT2017 dataset (Kermany et al., 2018).

The OCT-C8 dataset contains 24,000 OCT B-scan images from eight categories: age-related macular degeneration (AMD), choroidal neovascularization (CNV), central serous retinopathy (CSR), diabetic macular edema (DME), diabetic retinopathy (DR), Drusen, macular hole (MH), and Normal. Each category contains 3,000 images. Following the provided dataset split, OCT-C8 was divided into 18,400 training images, 2,800 validation images, and 2,800 testing images, with a balanced class distribution across the eight categories.

The OCT2017 dataset contains 84,484 OCT retinal images from four categories: CNV, DME, Drusen, and Normal. According to the public release, OCT2017 provides 83,484 training images and 1,000 testing images, with 250 testing images per category. Since no separate validation set is provided, a patient-level validation subset was split from the OCT2017 training set according to randomized patient identifiers, and the remaining training data were used for model training. The OCT2017 testing set was used for final performance evaluation. No patient demographics are released.

For both datasets, all images were resized to 224 × 224 and normalized using the ImageNet mean and standard deviation.

### 4.2 Evaluation metrics

To evaluate the classification performance of SLight-Net, we used accuracy, precision, recall (also referred to as sensitivity), F1-score, confusion matrix, and receiver operating characteristic (ROC) analysis. For multi-class evaluation, macro-averaged precision, recall, and F1-score were reported. One-vs-rest area under the curve (AUC) values were calculated for each category, and macro-average and micro-average AUC values were used to summarize the overall discriminative performance.

Accuracy measures the proportion of correctly classified samples among all test samples. It is calculated as follows:

Accuracy is calculated using Equation 33.

$$Acc=\frac{N_{correct}}{N}$$ (33)

where ( $N_{correct}$ ) denotes the number of correctly classified samples, and (N) denotes the total number of test samples. Accuracy is used as the primary metric in this study because both OCT-C8 and OCT2017 are standard classification benchmarks.

For each class, precision and recall are calculated to further analyze the class-wise recognition ability of the model.

Precision and recall are calculated using Equations 34, 35 respectively.

$$Precision_{c}=\frac{TP_{c}}{TP_{c}+FP_{c}}$$ (34)

$$Recall_{c}=\frac{TP_{c}}{TP_{c}+FN_{c}}$$ (35)

where ( $TP_{c}$ ), ( $FP_{c}$ ), and ( $FN_{c}$ ) denote the number of true positives, false positives, and false negatives for class (c), respectively. Precision reflects how many samples predicted as class (c) are correctly classified, while recall reflects how many real samples of class (c) are successfully recognized.

The F1-score is the harmonic mean of precision and recall, and it provides a balanced measurement when considering both false positives and false negatives:

The F1-score is calculated using Equation 36.

$$F1_{c}=\frac{2\times Precision_{c}\times Recall_{c}}{Precision_{c}+Recall_{c}}$$ (36)

where ( $F1_{c}$ ) denotes the F1-score of class (c). For multi-class evaluation, we report the macro-averaged precision, recall, and F1-score by averaging the corresponding class-wise values across all categories. Macro-averaged metrics treat each class equally and are therefore useful for evaluating performance on both balanced and imbalanced datasets.

Confusion matrices were used to visualize the distribution of predicted and ground-truth labels and to identify misclassification patterns among retinal disease categories.

ROC analysis was performed to evaluate class-wise discriminative ability. For multi-class classification, one-vs-rest ROC curves were generated, and the corresponding AUC value was calculated for each category. Macro-average and micro-average AUC values were also reported to summarize the overall discriminative performance.

To evaluate deployment efficiency, we reported the number of trainable parameters, estimated FLOPs, and end-to-end inference latency per OCT B-scan. These metrics were used to assess model compactness, computational cost, and practical inference efficiency.

For image-quality stratified analysis, an automatic quality score was computed using Tenengrad sharpness, image contrast, an intensity-based SNR proxy, and Laplacian variance. Images within each true class were divided into low-, medium-, and high-quality tertiles according to this score, and the corresponding tertiles were then merged across classes to reduce class-distribution bias.

### 4.3 Implementation details

All experiments were implemented using the PyTorch framework and conducted on a workstation equipped with a single NVIDIA GeForce RTX 4090 D GPU with 24 GB VRAM. Unless otherwise specified, experiments were conducted with a fixed random seed, and deterministic settings were enabled for CUDA/cuDNN where applicable. The same training pipeline was applied to both OCT-C8 and OCT 2017, with the classifier output dimension adjusted according to the number of categories in each dataset.

For repeated-seed evaluation on OCT-C8, the final SLight-Net model was trained using three random seeds: 42, 3,407, and 2024. The mean, standard deviation, and 95% confidence interval were calculated across the repeated runs. Inference latency was measured using an input tensor of size 1 × 3 × 224 × 224. For GPU inference, 100 warm-up iterations and 1,000 repeated runs were used. For CPU inference, 30 warm-up iterations and 200 repeated runs were used to reduce the total benchmarking time. The reported latency represents end-to-end inference time and therefore includes the FFT and inverse FFT operations in the FASE module.

SLight-Net was optimized using a two-stage training strategy. In Stage 1, the model was trained with data augmentation, including random horizontal flipping and random affine transformations with small rotation, translation, and scale perturbations. Label smoothing with a smoothing factor of 0.1 was applied to the cross-entropy loss. The auxiliary classification branch was used during training, and its loss weight was set to αaux = 0.4. The optimizer was AdamW with a weight decay of 1 × 10^(-4), and the initial learning rate was set to 1 × 10^(-3). A cosine annealing learning-rate scheduler was adopted. An exponential moving average (EMA) copy of the model weights was maintained during training with a decay factor of 0.999, and the best checkpoint was selected according to validation accuracy.

In Stage 2, the best checkpoint from Stage 1 was further fine-tuned using a lower learning rate of 1 × 10^(-5). Milder geometric augmentation was used in this stage, where horizontal flipping and slight translation/scale perturbations were retained, while rotation was removed. Batch Normalization statistics were frozen during Stage 2 fine-tuning to stabilize the learned feature distribution. EMA was also maintained in this stage, and the final evaluation result was obtained using the best checkpoint selected on the validation set.

A mini-batch size of 16 was used for training. For validation and testing, the batch size was set to 1. Automatic Mixed Precision (AMP) training was adopted during model optimization to reduce GPU memory consumption and improve training efficiency. Since FASE contains a Fourier-domain branch, FFT and inverse FFT operations in this branch were forced to single precision under AMP. This was done because half-precision arithmetic may be less stable for complex-valued Fourier coefficients with varying magnitudes, which can introduce rounding errors, gradient underflow/overflow, or unstable inverse FFT reconstruction.

### 4.4 The performance of SLight-Net

In this section, we report the classification performance of SLight-Net on OCT-C8 and OCT 2017, including quantitative comparison, confusion matrix, ROC analysis, image-quality stratified analysis, repeated-seed evaluation, and computational efficiency analysis.

**FIGURE 5:**
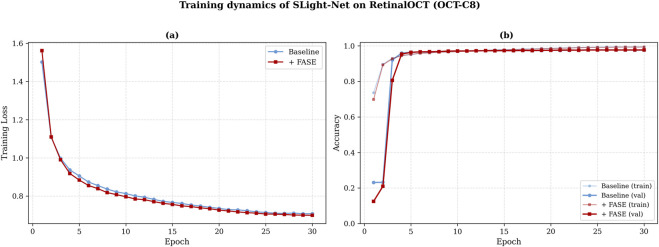
Learning curves of SLight-Net on the OCT-C8 dataset. **(a)** Training loss curve. **(b)** Training and validation accuracy curves.

Figure 5 presents the training loss and accuracy curves on OCT-C8. The loss decreases steadily and the training and validation accuracy rapidly converge to a stable high-performance level, indicating that the lightweight architecture can be effectively optimized under the adopted training strategy.

**FIGURE 6:**
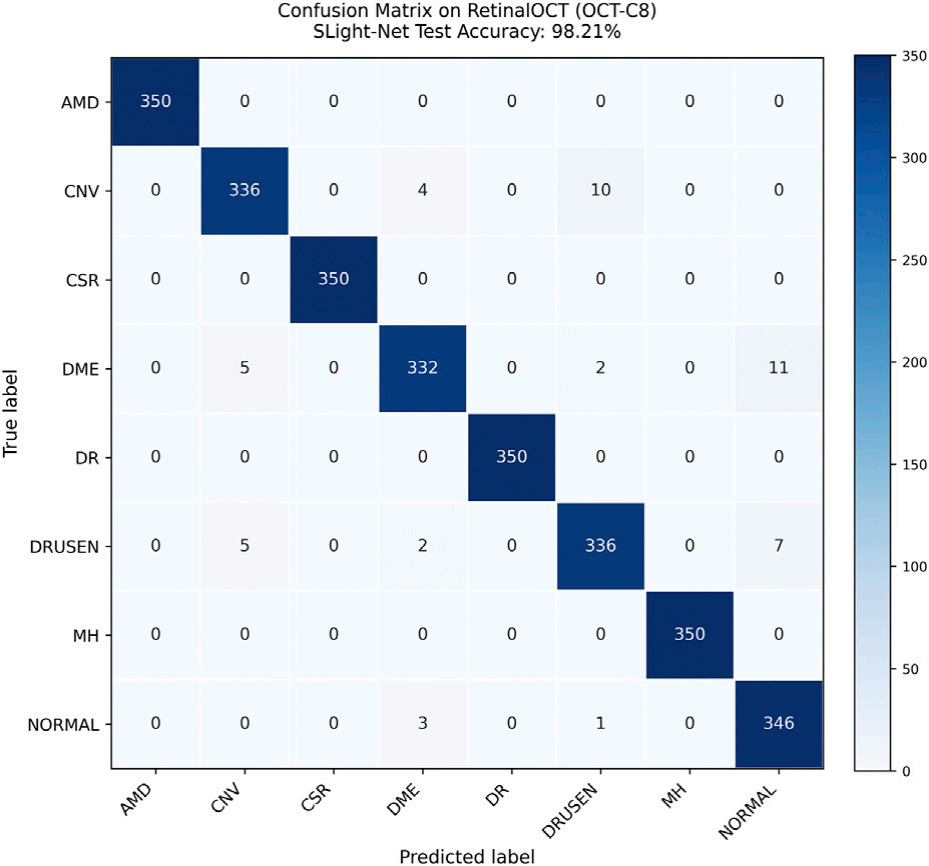
Confusion matrix of SLight-Net on the OCT-C8 dataset. The diagonal elements represent correctly classified samples, while the off-diagonal values indicate misclassified cases among different retinal disease categories.

To further analyze class-wise prediction behavior, Figure 6 shows the OCT-C8 confusion matrix. Most predictions are concentrated along the diagonal, indicating that SLight-Net correctly classifies the majority of test samples across the eight retinal categories. The remaining off-diagonal entries mainly occur among visually similar pathological categories, suggesting that residual errors are concentrated in difficult fine-grained cases rather than broad category separation.

For quantitative comparison, Table 1 summarizes the performance of SLight-Net and representative methods on the OCT-C8 dataset. SLight-Net achieves an accuracy of 98.21% with only 1.204M parameters, providing a favorable balance between classification performance and model compactness compared with both large generic backbones and lightweight networks.

**TABLE 1:** Performance comparison on the OCT-C8 dataset (%).

Method	Params (M)	Accuracy	Precision	Sensitivity	F1
ResNet-EdgeEn (Karthik and Mahadevappa, 2023)	—	92.40	93.00	92.00	92.00
HTC-Retina (Laouarem et al., 2024)	—	97.00	97.04	97.00	97.01
Swin-Poly Transformer (He et al., 2023)	—	97.11	97.13	97.11	97.10
VGG16 (Simonyan and Zisserman, 2015)	134.29	97.86	97.87	97.86	97.85
MSLI-Net (Qi et al., 2025)	89.69	96.72	96.72	96.72	96.72
Swin_Tiny (Liu et al., 2021)	29.00	97.75	97.75	97.75	97.75
ConvNeXtV2 (Woo et al., 2023)	28.60	97.39	97.40	97.39	97.39
WaveNet-SF (Cheng et al., 2026)	27.84	97.79	97.79	97.79	97.78
InceptionV3 (Szegedy et al., 2016)	23.83	97.96	97.97	97.96	97.96
ResNet50 (He et al., 2016)	23.52	97.61	97.61	97.61	97.60
EfficientNet-B3 (Tan and Le, 2019)	12.23	97.89	97.91	97.89	97.89
DenseNet121 (Huang et al., 2017)	6.96	97.89	97.90	97.89	97.89
GoogLeNet (Szegedy et al., 2015)	6.62	97.68	97.70	97.68	97.68
MobileNetV2 (Sandler et al., 2018)	3.50	96.46	96.49	96.46	96.46
MobileNetV3-Small (Howard et al., 2019)	2.54	96.71	96.75	96.71	96.71
ShuffleNetV2 (Ma et al., 2018)	2.28	97.38	97.40	97.42	97.41
SLight-Net (ours)	**1.20**	**98.21**	**98.23**	**98.21**	**98.21**

Bold values indicate the best performance among the compared methods.

Overall, the OCT-C8 results show that SLight-Net provides a favorable trade-off between diagnostic accuracy and model complexity. Although its parameter count is far smaller than that of many high-performing OCT models, it still achieves the best or highly competitive performance on the eight-class benchmark.

**FIGURE 7:**
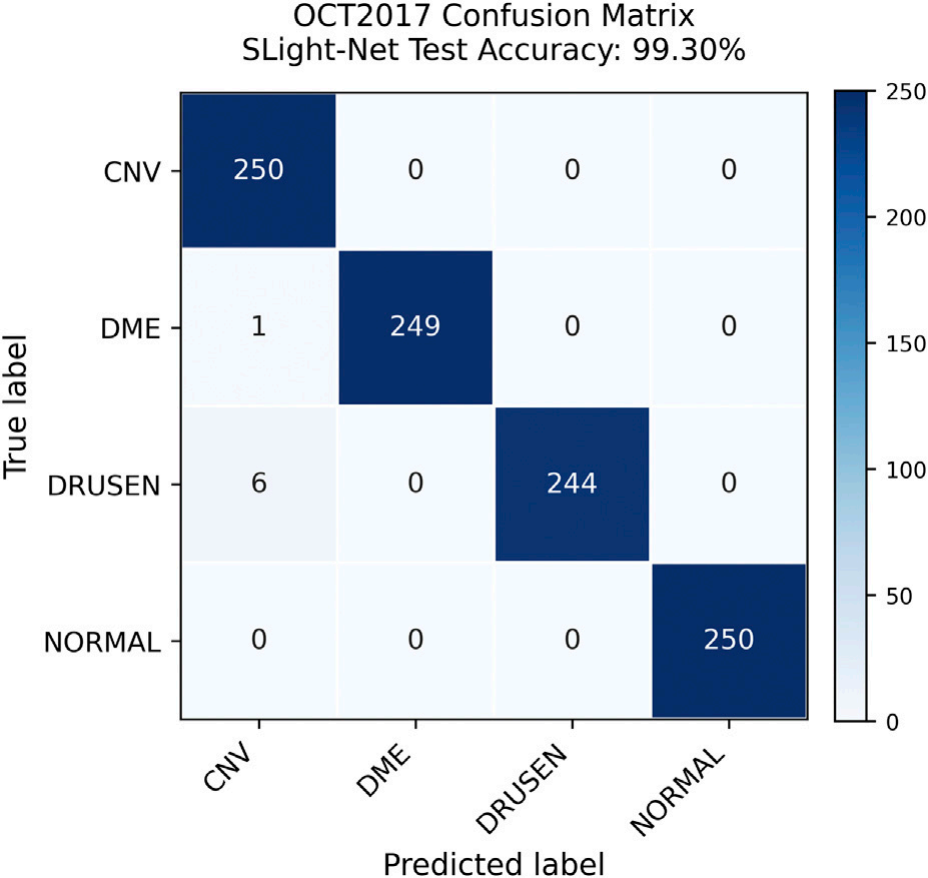
Confusion matrix of SLight-Net on the OCT2017 evaluation set. Most predictions are concentrated along the diagonal. Only a small number of DME and DRUSEN samples are misclassified as CNV, while CNV and NORMAL are correctly recognized in all test cases.

SLight-Net was evaluated on the four-class OCT2017 dataset and achieved 99.30% accuracy. Figure 7 shows the confusion matrix on the OCT2017 dataset, and Table 2 reports the quantitative comparison with representative methods. The OCT2017 confusion matrix shows that most predictions are concentrated along the diagonal. Only a small number of DME and DRUSEN samples are misclassified as CNV, whereas CNV and NORMAL are correctly recognized in all evaluated cases. The quantitative results in Table 2 further show that SLight-Net achieves the highest accuracy among the compared methods while retaining a compact architecture.

**TABLE 2:** Performance comparison on the OCT2017 dataset (%).

Method	Accuracy	Precision	Sensitivity	F1-score
EfficientNet-B3 (Tan and Le, 2019)	99.12	99.14	99.12	99.12
ResNet50 (He et al., 2016)	99.26	99.28	99.26	99.26
DenseNet121 (Huang et al., 2017)	99.26	99.27	99.26	99.26
InceptionV3 (Szegedy et al., 2016)	99.28	99.30	99.28	99.28
SLight-Net (ours)	**99.30**	**99.30**	**99.30**	**99.30**

Bold values indicate the best performance among the compared methods.

**FIGURE 8:**
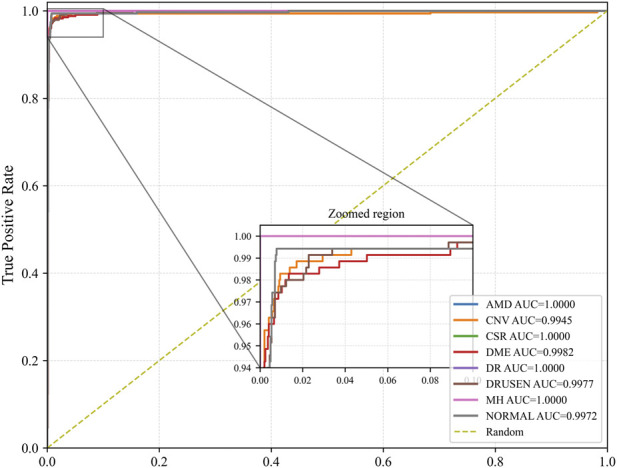
One-vs-rest ROC curves of SLight-Net on the OCT-C8 dataset. The macro-average AUC was 0.9985, and the micro-average AUC was 0.9988.

In addition to accuracy-based metrics, ROC analysis was performed for all OCT-C8 categories. As shown in Figure 8, SLight-Net achieved a macro-average one-vs-rest AUC of 0.9985 and a micro-average AUC of 0.9988. The class-wise AUC values were 1.0000 for AMD, 0.9945 for CNV, 1.0000 for CSR, 0.9982 for DME, 1.0000 for DR, 0.9977 for DRUSEN, 1.0000 for MH, and 0.9972 for NORMAL, indicating strong class-wise discrimination across all retinal categories.

To further examine the influence of image quality, we performed a class-balanced quality-stratified analysis on the OCT-C8 dataset. Quality groups were generated by dividing images within each true class into low-, medium-, and high-quality tertiles according to an automatic proxy quality score, followed by merging the corresponding tertiles across classes. The quality score was calculated using Tenengrad sharpness, image contrast, an intensity-based SNR proxy, and Laplacian variance. As shown in Table 3, SLight-Net maintained high performance across all quality groups, achieving accuracies of 97.76%, 98.40%, and 98.49% in the low-, medium-, and high-quality groups, respectively. The corresponding macro-F1 scores were 97.76%, 98.40%, and 98.49%, suggesting that SLight-Net remains robust across different image-quality levels.

**TABLE 3:** Class-balanced quality-stratified performance of SLight-Net on the OCT-C8 dataset.

Quality group	n	Accuracy (%)	Macro precision (%)	Macro recall (%)	Macro-F1 (%)
Low-quality	936	97.76	97.80	97.76	97.76
Medium-quality	936	98.40	98.41	98.40	98.40
High-quality	928	98.49	98.50	98.49	98.49

To evaluate training stability, the final SLight-Net model was trained and evaluated using three random seeds: 42, 3,407, and 2024. As shown in Table 4, SLight-Net achieved 98.24% ± 0.04% accuracy and 98.24% ± 0.04% macro-F1 on OCT-C8, with a 95% confidence interval of ±0.05% for accuracy. The 98.21% reported in Tables 1 and 5 corresponds to the primary single-run model (seed 42), whereas the 98.24% here is the mean over the three seeds; all runs fall within the reported confidence interval, confirming stable performance.

**TABLE 4:** Repeated-seed stability of SLight-Net on OCT-C8.

Metric	Mean ± SD (%)	95% CI (%)
Accuracy	98.24 ± 0.04	±0.05
Macro Precision	98.25 ± 0.04	±0.04
Macro Recall	98.24 ± 0.04	±0.05
Macro-F1	98.24 ± 0.04	±0.05

**TABLE 5:** Computational efficiency of SLight-Net.

Metric	Value
Parameters	1.204M
Estimated FLOPs	4.276G
CPU latency	33.11 ms/scan
GPU latency	2.94 ms/scan
Input size	1 × 3 × 224 × 224

To further evaluate deployment efficiency, we measured the computational cost and end-to-end inference latency of SLight-Net. As shown in Table 5, the final model contains 1.204M parameters and requires 4.276G estimated FLOPs for an input size of 1 × 3 × 224 × 224. The estimated FLOPs were obtained using THOP when available, with a Conv2d/Linear hook-based fallback, and should be interpreted as an approximate operation count. FFT/iFFT operations in the FASE module were not explicitly counted in the FLOPs estimation. Therefore, to directly reflect the runtime cost introduced by the Fourier-domain branch, we also reported end-to-end inference latency. The measured latency was 33.11 m per scan on CPU and 2.94 m per scan on an NVIDIA GeForce RTX 4090 D GPU. These results show that SLight-Net maintains practical inference efficiency while retaining the Fourier-domain operations in FASE.

### 4.5 Ablation study

To investigate the contribution of the proposed components, we conducted a progressive ablation study on the OCT-C8 dataset. Starting from a lightweight baseline without FASE and RLDA, we progressively introduced FASE and RLDA to evaluate their individual and combined effects.

Table 6 summarizes the progressive ablation results. Compared with the baseline, introducing FASE improves classification performance by enhancing spectral-spatial feature learning, and further adding RLDA provides additional gains through retinal depth-aware feature refinement. These results suggest that the improvement of SLight-Net is mainly attributed to the OCT-specific designs of FASE and RLDA rather than merely to an increase in model capacity. The ablation results in Table 6 are obtained from the primary single-run model (seed 42). Given the run-to-run standard deviation of ±0.04% for the full model (Table 4), the +0.15% gain from FASE and the +0.10% gain from RLDA exceed typical seed-level variation.

**TABLE 6:** Progressive ablation study of the proposed modules on the RetinalOCT (OCT-C8) dataset.

Configuration	Params (M)	Accuracy (%)	Δ
Baseline (without FASE and RLDA)	0.884	97.96	—
+ FASE	1.189	98.11	+0.15
+ FASE + RLDA (Full SLight-Net)	**1.204**	**98.21**	**+0.25**

Bold values indicate the results of the full SLight-Net configuration.

### 4.6 Qualitative visualization and error analysis

**FIGURE 9:**
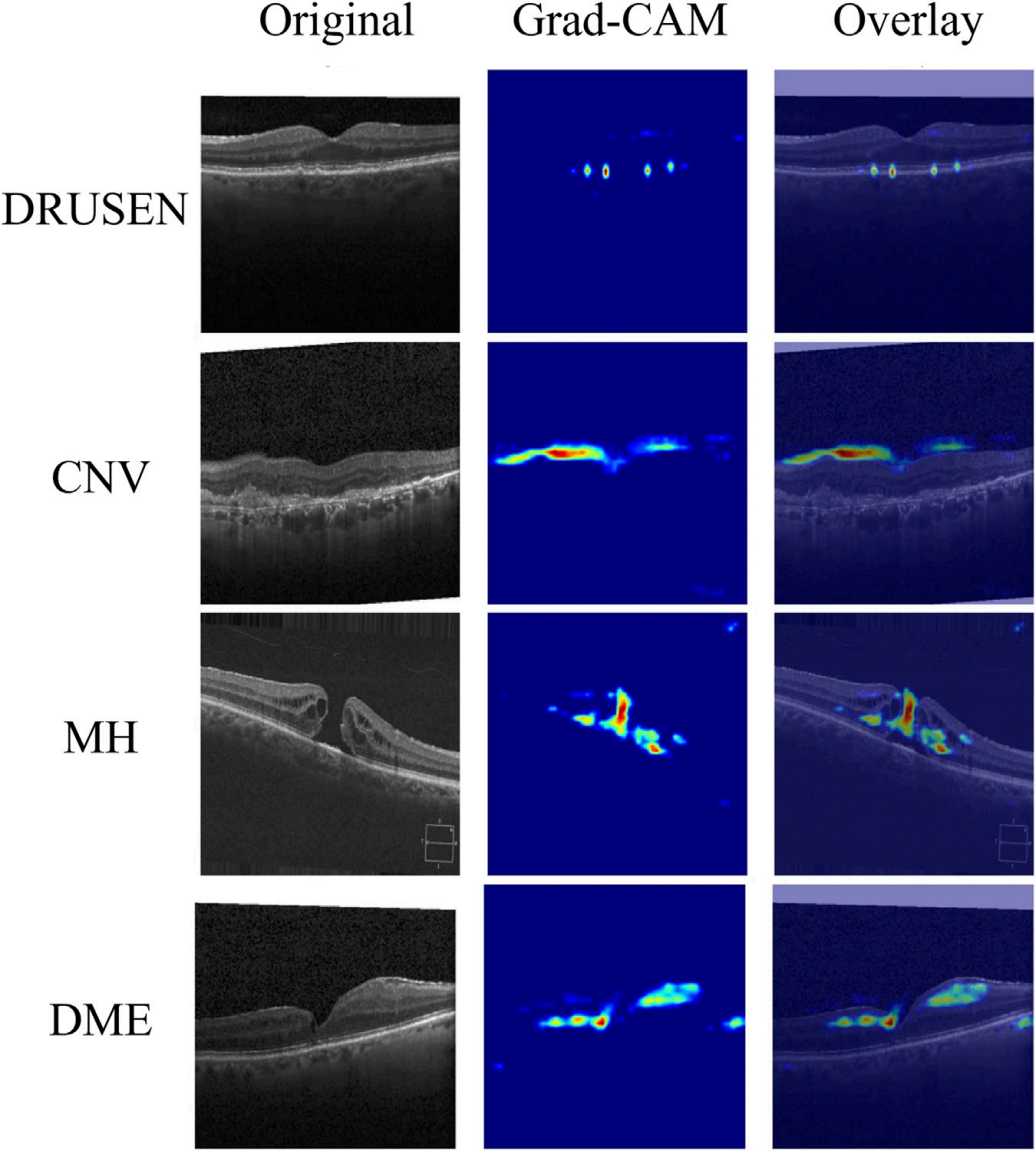
Grad-CAM visualizations of representative correctly classified OCT-C8 cases from four categories: DRUSEN, CNV, MH, and DME. Each row contains the original OCT B-scan, the Grad-CAM heatmap, and the overlay result. The activation regions are mainly located around disease-relevant retinal structures.

**FIGURE 10:**
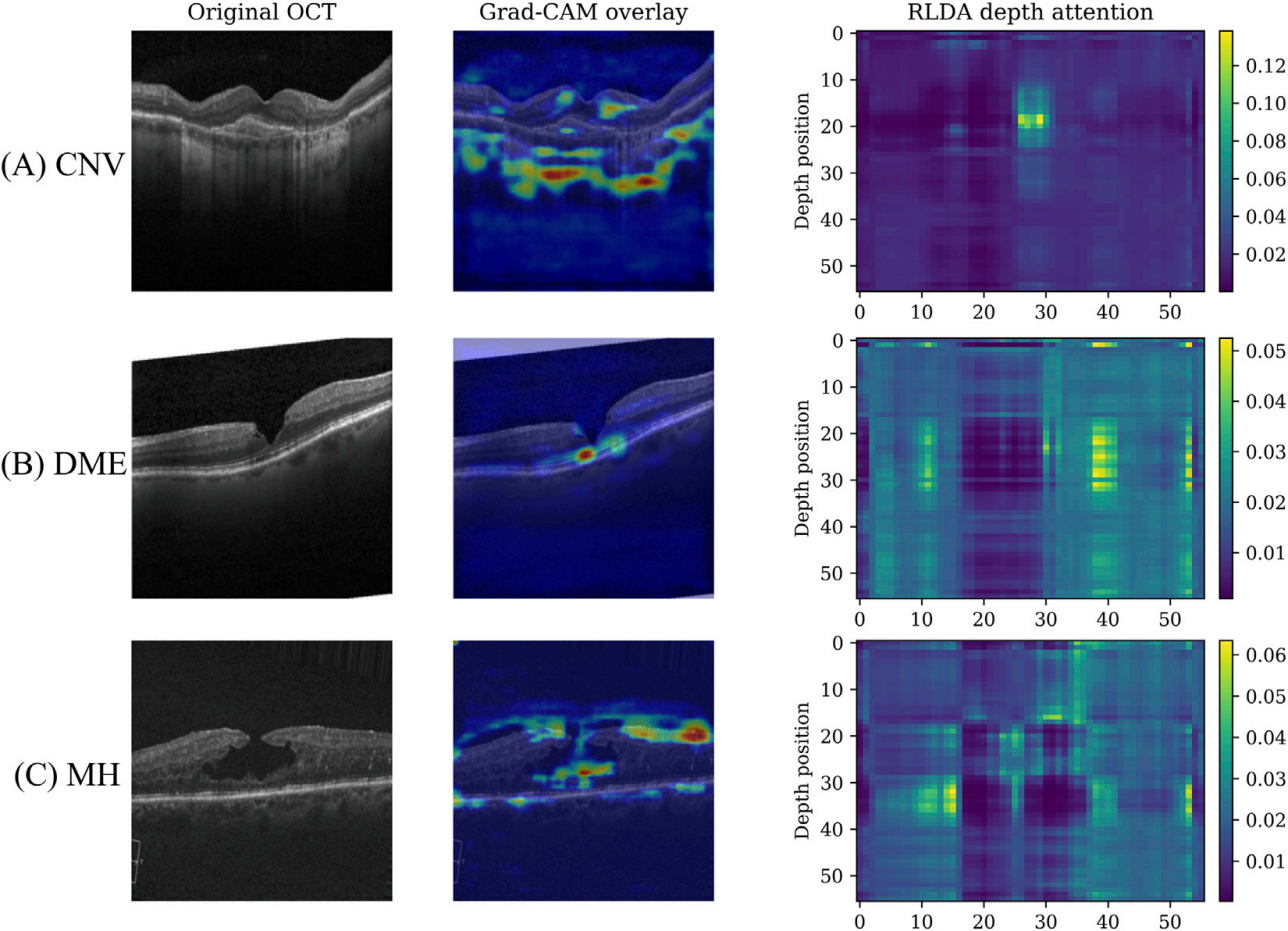
Grad-CAM and RLDA depth-attention visualizations of representative correctly classified OCT-C8 cases. **(A)** CNV, **(B)** DME, and **(C)** MH. Each row shows the original OCT B-scan, Grad-CAM overlay, and RLDA depth-attention map. The Grad-CAM results highlight disease-relevant retinal regions, while the RLDA maps show non-uniform depth-wise responses along the retinal depth direction.

To complement the quantitative evaluation, we further examined Grad-CAM visualizations on the OCT-C8 dataset. Figure 9 presents representative correctly classified cases from DRUSEN, CNV, macular hole, and diabetic macular edema. The activation regions are mainly located around disease-relevant retinal structures, such as RPE elevations, intraretinal cystic changes, and foveal defects, suggesting that SLight-Net tends to focus on anatomically meaningful regions during classification. To further examine the behavior of the proposed RLDA module, we visualized Grad-CAM overlays together with RLDA depth-attention maps for representative correctly classified cases, including CNV, DME, and MH. As shown in Figure 10, the Grad-CAM overlays highlight disease-relevant retinal regions, while the RLDA maps exhibit non-uniform responses along the retinal depth direction. These observations qualitatively suggest that RLDA recalibrates features according to depth-related retinal structural information and contributes to layer-aware feature refinement.

**FIGURE 11:**
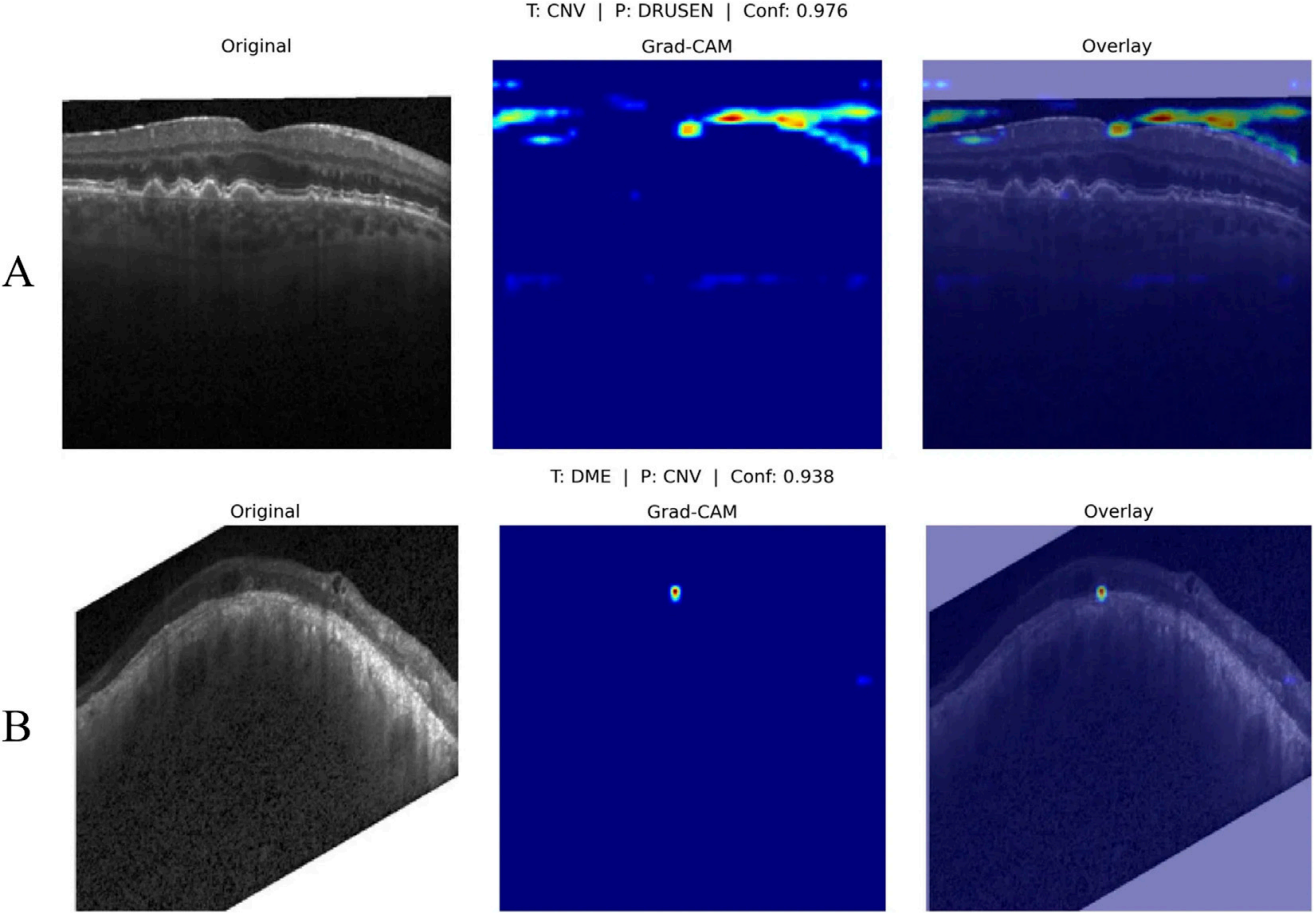
Grad-CAM visualizations of two representative misclassified cases. **(A)** A CNV case misclassified as DRUSEN, where the activation regions localize to elevated retinal structures that resemble drusen-like morphology. **(B)** A DME case misclassified as CNV, where low image quality and ambiguous retinal morphology make the pathological cues less distinctive. Each example shows the original OCT B-scan, the Grad-CAM heatmap, and the overlay result.

We also analyze representative misclassified samples to identify the remaining challenging cases. Figure 11 shows two difficult examples from OCT-C8. In the first case, a CNV image is misclassified as DRUSEN because the activated region overlaps with elevated retinal structures that resemble drusen-like morphology. In the second case, a DME image is misclassified as CNV, where low image quality and ambiguous retinal morphology make the pathological cues less distinctive.

These observations suggest that residual errors mainly arise from ambiguous morphology between visually similar categories and degraded image quality, rather than being dominated by a single systematic error pattern. Future improvements may benefit from lesion-level supervision, quality-aware preprocessing, volumetric OCT information, image-quality assessment, and uncertainty estimation.

## 5 Discussion

The experimental results show that SLight-Net achieves competitive performance on OCT-C8 while maintaining a compact parameter size. Its 98.21% accuracy with 1.204M parameters indicates that an OCT-specific lightweight architecture can provide a practical alternative to large generic backbones for retinal OCT classification. On OCT 2017, SLight-Net achieved 99.30% accuracy, further suggesting that the proposed model maintains strong performance under a different class setting.

The effectiveness of SLight-Net is mainly attributable to the complementary design of FASE and RLDA. FASE integrates local convolution, dilated contextual modeling, and Fourier-domain modulation to capture subtle pathological textures and retinal boundary information. RLDA further introduces a depth-axis anatomical prior that reflects the laminar organization of retinal OCT B-scans. Together, these modules allow the model to exploit both OCT signal characteristics and retinal structural regularity without substantially increasing model scale.

Although SLight-Net achieved favorable results on both datasets, its generalization to broader clinical settings remains an important consideration. OCT-C8 and OCT2017 are public retrospective benchmarks and may not fully capture the heterogeneity of real-world ophthalmic practice. Differences in OCT devices, imaging protocols, clinical centers, image quality, patient populations, and annotation practice may affect model performance. Since additional multi-center external validation datasets with complete device and demographic information were not available in this study, the current results should be regarded as benchmark-level evidence rather than definitive proof of real-world clinical generalization. In addition, although the quality-stratified analysis suggests relatively stable performance across different image-quality levels, the quality groups were defined using automatic image-quality proxies rather than manual clinical quality grading.

From a clinical workflow perspective, SLight-Net is intended to serve as a lightweight computer-aided screening and triage tool rather than a replacement for ophthalmologists. In practical OCT workflows, the model could be integrated after image acquisition to provide rapid image-level probability estimates, flag suspicious scans, and help prioritize cases requiring specialist review. This may be useful in high-throughput screening scenarios or resource-limited settings where large numbers of OCT B-scans need to be reviewed. The compact parameter size and measured inference latency of 33.11 m per scan on CPU and 2.94 m per scan on an NVIDIA GeForce RTX 4090 D GPU further support its potential for rapid OCT B-scan screening. Nevertheless, the model should be used as an assistive tool under physician oversight, and prospective clinical validation is required before real-world diagnostic use. This lightweight OCT-specific design is also consistent with recent discussions on AI-assisted ophthalmic practice and reliable medical-image analysis (Xu and Yang, 2023; Wu et al., 2024; Wan et al., 2022; Shao et al., 2025; Cao et al., 2025).

This study has several limitations. First, the experiments were conducted on public retrospective OCT datasets, and no prospective multi-center clinical validation was performed. Second, SLight-Net currently performs image-level classification on individual two-dimensional B-scans, whereas clinical OCT interpretation often relies on volumetric information across adjacent B-scans. Third, although Grad-CAM visualizations, RLDA depth-attention maps, and error analysis provide preliminary interpretability, the model does not include an explicit mechanism for dynamic image-quality assessment, uncertainty estimation, or quality-aware rejection. Low signal-to-noise ratio, motion artifacts, severe speckle noise, or blurred retinal boundaries may still reduce classification reliability. Fourth, complete patient-level demographic information, OCT device information, and acquisition metadata were not available in the public datasets, preventing subgroup analyses by patient population, scanner type, or clinical center. Future work will focus on prospective multi-center validation, device-independent evaluation, volumetric OCT modeling, quality-aware preprocessing, and clinically grounded lesion-aware interpretation.

## 6 Conclusion

In this paper, we proposed SLight-Net, a compact and effective convolutional neural network for retinal OCT disease classification. Unlike many high-performing methods that rely on large generic backbones, SLight-Net is specifically designed around the structural and signal characteristics of OCT images. The final model contains only 1.204M parameters, making it suitable for deployment-oriented retinal screening scenarios where both accuracy and model efficiency are important.

The proposed framework introduces two OCT-specific core modules. First, the Frequency-Aware Spectral-Spatial Encoder (FASE) integrates local convolution, dilated contextual modeling, and Fourier-domain modulation through a selective fusion mechanism, enabling the model to capture fine retinal boundaries, broader anatomical context, and subtle texture cues. Second, the Retinal Layer Depth Attention (RLDA) module incorporates the depth-axis anatomical prior of retinal OCT images by combining learnable depth bias, vertical depth-wise modeling, and cross-depth attention. These two modules provide complementary feature modeling capabilities for lightweight OCT classification.

Experiments on OCT-C8 show that SLight-Net achieves 98.21% accuracy with only 1.204M parameters, and the supporting OCT2017 evaluation reaches 99.30% accuracy. Compared with representative CNN, Transformer, and lightweight models, SLight-Net achieves a favorable balance between classification performance and parameter efficiency on the main benchmark.

Overall, SLight-Net demonstrates that a carefully designed lightweight CNN can achieve competitive OCT classification performance without relying on a large-scale backbone. By combining frequency-aware spectral-spatial representation with retinal layer-depth prior modeling, the proposed method provides an efficient and interpretable solution for automated OCT-based retinal disease classification. In future work, we will further validate the proposed model on larger multi-center clinical datasets, extend it to volume-level OCT analysis, and explore more clinically interpretable lesion-aware learning strategies.

## Data Availability

The original contributions presented in the study are included in the article/supplementary material, further inquiries can be directed to the corresponding authors.
